# Evaluation of [^18^F]AlF-EMP-105 for Molecular Imaging of C-Met

**DOI:** 10.3390/pharmaceutics15071915

**Published:** 2023-07-10

**Authors:** Jin Hui Teh, Ala Amgheib, Ruisi Fu, Chris Barnes, Joel Abrahams, Ali Ashek, Ning Wang, Zixuan Yang, Muneera Mansoorudeen, Nicholas J. Long, Eric O. Aboagye

**Affiliations:** 1 Department of Chemistry, Molecular Sciences Research Hub, Imperial College London, London W12 0BZ, UK; jin.teh15@imperial.ac.uk (J.H.T.); n.long@imperial.ac.uk (N.J.L.); 2 Department of Surgery and Cancer, Imperial Centre for Translational and Experimental Medicine, Imperial College London, London W12 0NN, UK; a.amgheib@imperial.ac.uk (A.A.); ruisi.fu14@imperial.ac.uk (R.F.); chris.barnes@imperial.ac.uk (C.B.); joel.abrahams@imperial.ac.uk (J.A.); m.ashek@imperial.ac.uk (A.A.); n.wang16@imperial.ac.uk (N.W.); zixuan.yang18@imperial.ac.uk (Z.Y.); m.mansoorudeen22@imperial.ac.uk (M.M.)

**Keywords:** c-Met, PET/CT, [^18^F]AlF, tyrosine kinase receptors

## Abstract

C-Met is a receptor tyrosine kinase that is overexpressed in a range of different cancer types, and has been identified as a potential biomarker for cancer imaging and therapy. Previously, a ^68^Ga-labelled peptide, [^68^Ga]Ga-EMP-100, has shown promise for imaging c-Met in renal cell carcinoma in humans. Herein, we report the synthesis and preliminary biological evaluation of an [^18^F]AlF-labelled analogue, [^18^F]AlF-EMP-105, for c-Met imaging by positron emission tomography. EMP-105 was radiolabelled using the aluminium-[^18^F]fluoride method with 46 ± 2% RCY and >95% RCP in 35–40 min. In vitro evaluation showed that [^18^F]AlF-EMP-105 has a high specificity for c-Met-expressing cells. Radioactive metabolite analysis at 5 and 30 min post-injection revealed that [^18^F]AlF-EMP-105 has good blood stability, but undergoes transformation—transchelation, defluorination or demetallation—in the liver and kidneys. PET imaging in non-tumour-bearing mice showed high radioactive accumulation in the kidneys, bladder and urine, demonstrating that the tracer is cleared predominantly as [^18^F]fluoride by the renal system. With its high specificity for c-Met expressing cells, [^18^F]AlF-EMP-105 shows promise as a potential diagnostic tool for imaging cancer.

## 1. Introduction

C-mesenchymal-epithelial transcription factor (c-Met) is a receptor tyrosine kinase that is activated by the hepatocyte growth factor (HGF). The aberrant activation of c-Met and dysregulation of the MET/HGF pathway can lead to cancer cell proliferation, tumour growth, and metastasis [1,2,3,4]. Multiple studies have shown that c-Met is overexpressed in a range of different cancers, including colorectal cancer [5], breast [6], lung [7], pancreatic [8], prostate [9], gastric [10], renal [11], ovarian cancer [12], melanoma [13], nervous system malignancies [14], and pediatric tumours [15], making it a valuable target for cancer therapy. As a result, since 2010, four small molecule drugs—crizotinib, cabozantinib, tepotinib and capmatinib—have been approved by the United States Food and Drug Administration (US FDA) for cancer treatment by c-Met inhibition [1,16,17]. Apart from these, accelerated approval and breakthrough therapy designation (BTD) were granted, respectively, for Amivantamab and telisotuzumab vedotin for the treatment of c-Met-overexpressing non-small cell lung cancer (NSCLC) [18,19], signifying the importance of c-Met as a biomarker. The MET/HGF pathway also positively regulates cancer stem cell enrichment and tumour aggressiveness due to stem cell-related resistance to therapy [14,20]. In this regard, the development of probes for c-Met imaging would further aid in improving diagnosis, patient selection, and assessment of patient response to c-Met targeted therapy.

**Figure 1:**
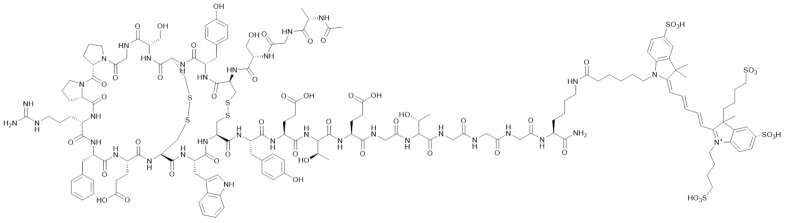
Structure of EMI-137. Peptide sequence: AGSCYCSGPPRFECWCYETEGT-Cy5.

A diverse range of probes for c-Met detection have been reported based on different imaging modalities, including fluorescence [21,22], magnetic resonance imaging (MRI) [23,24,25,26], and positron emission tomography (PET) [1]. Notably, a 26-amino acid cyclic peptide, GE-137 (now known as EMI-137, Figure 1), has been reported to be safe and effective for the detection of c-Met in a fluorescence-guided colonoscopy study [22], intra-operative colonic tumour mapping [27], endoscopy [28], and image-guided surgery in human patients [29,30]. Compared to these imaging modalities, PET offers several advantages, including high sensitivity, real-time imaging, and unlimited penetration depth, enabling the non-invasive detection of c-Met in vivo. A number of PET probes for imaging c-Met have been reported, and these are based on the HGF ligand, antibodies, peptides, and small molecules [1]. Although routine clinical use of PET tracers for c-Met detection has yet to be reported, peptide probes based on the structure of EMI-137 appear most promising. Notably, a gallium-68 (^68^Ga, *t*_1/2_ = 68 min, *E*_β+, max_ = 1.9 MeV) analogue, [^68^Ga]Ga-EMP-100, showed favourable results in detecting metastatic renal cell carcinoma in recent human trials (SUV_max_ of 4.35, SUV_mean_ of 2.52) [31]. A fluorine-18 (^18^F)-labelled analogue of EMI-137, [^18^F]F-AH113804, has also been reported by Appitha et al. to show promise for imaging basal-like breast cancer (BLBC) in mice [2].

Due to its ideal physical properties (^18^F, *t*_1/2_ = 110 min, *E*_β+, max_ = 0.64 MeV), fluorine-18 remains the most widely used isotope in PET imaging [32,33]. However, traditional methods to incorporate the ^18^F isotope, such as those employed by Appitha et al., typically make use of nucleophilic substitution reactions, which require specialist production equipment and expertise in ^18^F-fluorination chemistry [34,35]. Thus, we aim to develop a facile method to access an ^18^F-labelled analogue of EMI-137. We hypothesize that this could be achieved using the aluminium-[^18^F]fluoride ([^18^F]AlF) method reported by McBride et al., which combines the convenience of radiometal-based labelling, the favourable decay characteristics of fluorine-18 [36,37], and has the potential to be formulated into a kit [38]. With a recent study showing that [^18^F]AlF-NOTA-Octreotide outperforms its ^68^Ga counterpart for imaging SSTR2 [39], the results from this study could offer a valuable alternative to [^68^Ga]Ga-EMP-100 for imaging c-Met.

In this study, we aim to develop an [^18^F]AlF-labelled c-Met-targeting agent, [^18^F]AlF-EMP-105, as an alternative imaging agent for cancer. It is expected that [^18^F]AlF-EMP-105 shares similar biodistribution kinetics and radiation dosimetry as [^68^Ga]Ga-EMP-100. The metabolism of ^68^Ga- and [^18^F]AlF-labelled tracers is often not reported; instead, a simple EDTA method to assert potential for liver/kidney transchelation is conducted. However, this does not always fully translate to in vivo conditions. Failure to translate to in vivo conditions can be explained in large part by the overexpression of Cu-dependent proteins, including superoxide dismutase, caeruloplasmin and metallothionein, in the liver and kidneys. As these proteins can impact the disposition of tracers, we elaborate on this property of [^18^F]AlF-EMP-105.

## 2. Materials and Methods

### 2.1. General Considerations

EMP-105 and EMI-137 were kind gifts from Dr. Alex Gibson, Dr. Christophe Portal and Niall Swanwick of Edinburgh Molecular Imaging Ltd. (Edinburgh, UK). All reagents and solvents were used as purchased from commercial sources unless otherwise stated. HPLC grade acetonitrile, trifluoroacetic acid, DMSO and ethanol were purchased from Sigma-Aldrich (St. Louis, MO, USA). Solid phase extraction (SPE) cartridges were purchased from Waters (Wilmslow, UK). 

Analytical radio-HPLC chromatograms were obtained using an Agilent 1200 series instrument equipped with a flow-ram detector (Lablogic, Sheffield, UK), and integrated using Laura 6 software (Lablogic, Sheffield, UK). Column: phenomenex Aeris^TM^ 3.6 µm WIDEPORE C4 200 Å. Mobile phase: 0.1% TFA H_2_O:MeCN 95:5 *v/v* to 5:95 *v/v*, 0.8 mL/min flow rate.

For metabolite analysis, radio-HPLC chromatograms were obtained using an Agilent 1100 system equipped with an in-line posiRAM metabolite detector (Lablogic, Sheffield, UK). The same column and mobile phase were used.

### 2.2. Radiosynthesis of [^18^F]AlF-EMP-105

Prior to usage, ^18^F^−^ was trapped on a Sep-PAK Accell Plus QMA light cartridge (Cl^−^ form, Waters, cat. No. WAT023525), and eluted with 0.9% *w:v* NaCl solution.

EMP-105 in DMSO (50 nmol, 5 µL), 2 mM AlCl_3_ in 0.5 M NaOAc at pH 4.2 (25 µL, 50 nmol), purified [^18^F]fluoride (300–400 MBq, 150 µL) and DMSO (200 µL) were mixed and incubated at 100 °C for 20 min. Upon completion, the reaction was diluted with 0.1% TFA in water (15 mL), trapped on a Sep-Pak tC2 Plus Light Cartridge (145 mg), washed with 3 mL water, and eluted with ethanol (500 µL) in fractions (2–3 drops per fraction). The fraction with the highest radioactive concentration was used (typically fraction 2). Here, *t*_R_ [^18^F]AlF-EMP-105 = 8 min 17 s. EMP-105 was used to generate the calibration curve for molar activity calculations instead of [^19^F]AlF-EMP-105 (ESI, Figure S5).

### 2.3. Stability Tests

A fraction of purified [^18^F]AlF-EMP-105 (10 MBq in ~10 µL EtOH) was incubated, respectively, in 1000 µL of EtOH, PBS, RPMI, human serum and EDTA (0.02% in 0.5 mM DPBS) at 37 °C, and analysed by RP-HPLC at 1, 2, 3, and 4 h time points. For stability towards radiolysis, aliquots of >2 GBq of purified [^18^F]AlF-EMP-105 eluted from the cartridge in ethanol (75% in 10 mM H_3_P0_4_) or formulated in PBS solution containing 7.5% ethanol and 5 mg/mL (10 mL) of 4-aminobenzoic acid (PABA) as radioprotectant were analysed by RP-HPLC.

### 2.4. Cell Culture

H1975 (non-small cell lung cancer), HEPG2a (hepatocellular carcinoma), HT29 (colorectal cancer) were purchased from ATCC. OE21 (oesophageal squamous cell carcinoma) was obtained from Prof George Hanna. H1975 and OE21 cells were maintained in RPMI-1640 media (Sigma-Aldrich) supplemented with 10% fetal calf serum (Sigma-Aldrich), 1% L-glutamine, and 2% penicillin/streptomycin (Sigma-Aldrich). HEPG2a and HT29 cells were cultured in DMEM (Sigma-Aldrich) containing 10% fetal calf serum, 1% L-glutamine, and 2% penicillin/streptomycin. All cell lines were cultured at 37 °C and 5% CO_2_.

### 2.5. Flow Cytometry 

Approximately 500,000 cells were seeded in 6-well plates and cultured for 24 h at 37 °C and 5% CO_2_. Cells were washed with warm PBS and incubated with 50 nM EMI-137 for 1 h at 37 °C and 5% CO_2._ Subsequently, cells were washed with ice-cold PBS three times prior to sample acquisition via FACS Canto flow cytometer (Becton Dickinson Immunocytometry Systems, Franklin Lakes, NJ, USA) with FACS Diva Software version 4.0.2. The obtained data were analysed using FlowJo software v7.6 (FlowJo, LLC, Ashland, OR, USA). Unstained controls were used to define gates and adjust fluorescence compensation. 

### 2.6. Internalisation Assay 

H1975 cells were seeded in a 6-well plate at a seeding density of 500,000 cells/well, 24 h prior to performing internalisation assay. Cells were washed with warm PBS and incubated with 50 nM EMI-137 for 30 min or 1 h at either 4 °C or 37 °C, respectively. Subsequently, cells were washed with ice-cold PBS three times. To determine internalisation levels at 4 °C, surface-bound EMI-137 was removed by washing the cells with 50 mM of glycine prepared in 150 mM NaCl (Sigma) for 5 min followed by three washes with ice-cold PBS. To evaluate the internalisation dynamics of EMI-137, the incubation media (with EMI-137 at 4 °C for 30 min) were replaced with warm fresh media and incubated for 5, 15, and 30 min at 37 °C and 5% CO_2_. Cells were then washed with glycine as described above. Samples were acquired with an FACS Canto flow cytometer (Becton Dickinson Immunocytometry Systems). Data were presented as mean Fluorescence Intensity (MFI). The internalised fraction was determined by subtracting the surface-bound EMI-137 from total EMI-137. The percentage of internalised fraction was calculated by dividing the amount of internalised EMI-137 by total-bound EMI-137 multiplied by 100%. Unstained controls were used to define gates and adjust fluorescence compensation. 

### 2.7. In Vitro Uptake of [^18^F]AlF-EMP-105

Cells were seeded at appropriate densities (~500,000 cells/well in a 6-well plate) and allowed to attach overnight. On the day of uptake, cells were washed three times with warm PBS and incubated with 1 mL of fresh media containing approximately 0.74 MBq of [^18^F]AlF-EMP-105, with or without 100× molar equivalent of EMI-137 (blocking compound), in a humidified condition with 5% CO_2_ at 37 °C for 60 min. After 60 min of incubation, the cells were washed with ice-cold PBS (3 times), then lysed in 1 mL of RIPA buffer for 10 min on ice. Following this step, radioactivity from 800 µL of lysate from each sample was counted on a WIZARD2^®^ Automatic Gamma Counter. To determine the specificity of uptake, data were expressed as a percentage of radioactivity incorporated into cells, in untreated cells compared to cells blocked with EMI-137.

### 2.8. In Vivo PET Imaging

All animal experiments were performed by licensed investigators in accordance with the UK Home Office Guidance on the Operation of the Animal (Scientific Procedures) Act (ASPA) 1986 (HMSO, London, UK, 1990) and within the guidelines set out by the UK National Cancer Research Institute Committee on Welfare of Animals in Cancer Research [40]. Studies were conducted under Project License number 1780337.

Female BALB/c mice (6–8 weeks old) were anaesthetized with 2% isoflurane/O_2_. Imaging was performed using a Siemens Inveon small-animal multimodality PET/CT system (Siemens Medical Solutions USA, Inc., Knoxville, TN, USA). After completion of the CT scan, [^18^F]AlF-EMP-105 (4.6 ± 1.2 MBq) was injected intravenously via the lateral tail vein. Dynamic emission scans were acquired in list-mode format for 60 min. Image data were processed as 0.5 mm sinogram bins and 33 time-frames, and reconstructed using the 2D-ordered subsets expectation maximization (2D-OSEM) algorithm with CT-based attenuation correction. The following frame durations were used: 12 × 5 s, 4 × 15 s, 6 × 30 s, and 11 × 300 s. Images were analysed using Inveon Research Workplace software (Siemens Healthcare Molecular Imaging, Knoxville, TN, USA). PET and CT images were co-registered and used to draw 3-dimensional regions of interests (ROIs) over tissues to obtain time–activity curves (TACs). Decay-corrected tissue time versus radioactivity curves (TACs) were generated and normalized to whole-body activity to obtain normalized uptake values (NUVs) [41].

### 2.9. Metabolite Analysis

In female BALB/c mice at 5 and 30 min p.i. of [^18^F]AlF-NOTA-EMP-105, four key tissues (blood plasma, liver, kidney and urine) were analysed for radioactive metabolites by radio-HPLC. The retention time of free [^18^F]fluoride and the parent compound [^18^F]AlF-EMP-105 were determined by injecting a pure sample of each onto the metabolite radio-HPLC system. 

The liver and kidneys were excised, and homogenised in ice cold MeCN:H_2_O (1 mL, 1:2 *v/v*) using a Precellys tissue homogeniser fitted with a Cryolys cooling module (Stretton Scientific Ltd., Derbyshire, UK). The homogenate was centrifuged (13,000× *g*, 5 min), the supernatant was removed, filtered (0.22 µm syringe filter) and diluted in water prior to RP-HPLC analysis. Urine was diluted in water and filtered prior to HPLC analysis. Plasma was obtained from whole blood by centrifugation (2000× *g*, 10 min) to separate the blood cells from the plasma. Plasma were precipitated with ice cold MeCN:H_2_O (1 mL, 1:2 *v/v*), and centrifuged (13,000× *g*, 5 min) to pellet the proteins. The supernatant was filtered and diluted in water for radio-HPLC analysis. The HPLC injection loop was washed with MeCN:H_2_O 1:1 *v/v* (1 mL) and then 5:95 *v/v* (1 mL) between each injection. The extraction efficiency from each tissue sample was determined by counting the activity (counts per minute, CPM) of a small aliquot (20 µL) of the supernatant of a known volume and the whole protein pellet, in a γ-counter. 

### 2.10. Ex Vivo Biodistribution 

Ex vivo biodistribution studies were carried out in the same animals that underwent PET imaging. Briefly, immediately after the PET scan, mice were sacrificed by exsanguination via cardiac puncture and selected tissues were dissected and counted in a gamma-counter (Wizard 2480 Automatic Gamma Counter, Perkin Elmer, Waltham, MA, USA). The radiotracer biodistribution was expressed as a percentage of injected dose per gram of tissue (%ID/g).

### 2.11. Statistical Analysis

Data for radiolabelling were presented as mean values ± standard deviation (SD). In vitro uptake data were presented as mean ± standard error (SEM). Unpaired two-tailed *t*-tests from GraphPad Prism 7.0 were used to determine the significance in the experiments. Differences were considered statistically significant when *p* < 0.05.

## 3. Results

### 3.1. Radiochemistry

**Figure 2:**
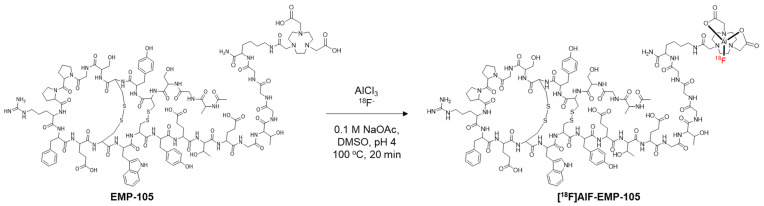
[^18^F]AlF-labelling of EMP-105.

The conditions for the radiolabelling of EMP-105 are shown in Figure 2. For radiolabelling by the [^18^F]AlF method, the use of an organic co-solvent has been shown to increase reaction yield [42]. In this reaction, DMSO was chosen as the co-solvent because EMP-105 is fully soluble. In contrast, the peptide was only sparingly soluble in most organic solvents such as MeCN, methanol and ethanol.

To maximise the molar activity (A_m_) and isolated activity of [^18^F]AlF-EMP-105, optimisation of the amount of precursor used in the reaction was conducted (Table 1). Expectedly, both radiochemical conversion (RCC) and radiochemical yield (RCY) increased with increasing amounts of precursor. The moderate RCYs obtained in this study (20–50%) were consistent with reported yields for [^18^F]AlF-labelling using NOTA-chelators, which ranged from 25 to 58%, and were also shown to increase with increasing precursor amount [36,43,44]. Upon purification by solid-phase extraction, [^18^F]AlF-EMP-105 was obtained in >95% radiochemical purity (RCP) (ESI, Figures S1 and S2). Since 50 nmol of precursor gave the maximum A_m_ without significantly compromising reaction yield, further studies were conducted on this scale. A test for residual solvent was not assessed at this stage of the development, which will be included in future studies.

[^18^F]AlF-EMP-105 showed excellent stability in PBS, RPMI and human serum ex vivo at 37 °C, with 95%, 97% and 92% of the compound remaining intact in the respective media after four hours (ESI, Figure S3 and Table S1). Transchelation of the [^18^F]AlF^2+^ complex was also not observed when incubated with 100 equivalence of EDTA, with 98% of the tracer remaining intact after 4 h (ESI, Figure S3 and Table S1). 

### 3.2. In Vitro Uptake 

**Figure 3:**
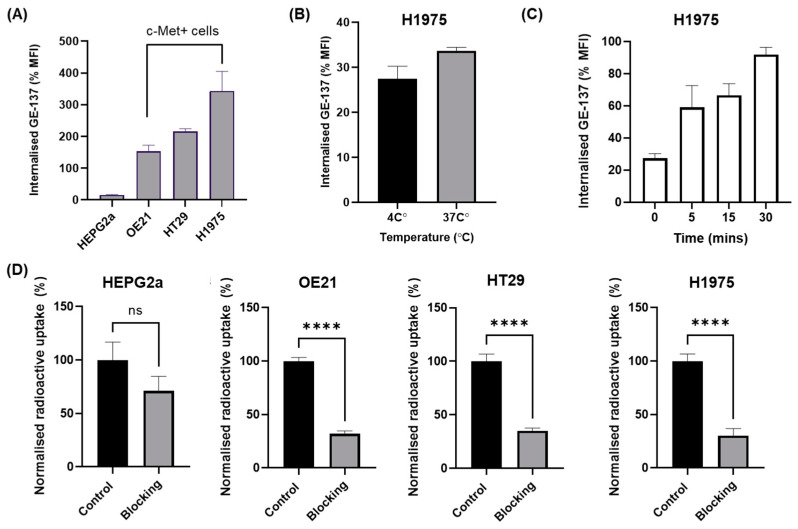
In vitro fluorescent uptake results of EMI-137, determined by flow cytometry (**A**–**C**). (**A**) Uptake of EMI-137 after incubation at 37 °C for 1 h, showing OE21, HT29 and H1975 as c-Met positive cell lines. (**B**) Uptake of EMI-137 in H1975 after incubation for 30 min at 4 and 37 °C. (**C**) Internalisation of EMI-137 in H1975 at 0, 5, 15 and 30 min at 37 °C. (**D**) Radioactive uptake data of [^18^F]AlF-EMP-105 after incubation at 37 °C for 1 h. Blocking studies were conducted by incubation with 100x excess of EMI-137. Data are presented as mean ± SEM. ns: No Significance; **** *p* < 0.0001.

Four cancer cell lines were chosen to evaluate the specificity of [^18^F]AlF-EMP-105 for c-Met (Figure 3). The level of expression of c-Met for each cell line was first evaluated by flow cytometry by incubation with EMI-137 (Figure 3A). High uptakes of EMI-137 were observed for OE21, HT29 and H1975, whereas HEPG2a showed a low uptake and was used as a negative control. Although EMI-137 showed a slightly higher uptake at 37 °C than 4 °C, the difference was not significant (Figure 3B). Expectedly, internalisation of EMI-137 in H1975 cells increased with time (Figure 3C).

Upon identification of the c-Met-expressing cell lines by EMI-137, the radioactive uptake of [^18^F]AlF-EMP-105 was evaluated (Figure 3D). Gratifyingly, the difference in uptake of [^18^F]AlF-EMP-105 with and without blocking in HEPG2a cells was not significant. In comparison, all c-Met-positive cell lines showed a significant decrease in uptake of [^18^F]AlF-EMP-105 when blocked with 100-fold of EMI-137. 

### 3.3. Metabolite Analysis

**Figure 4:**
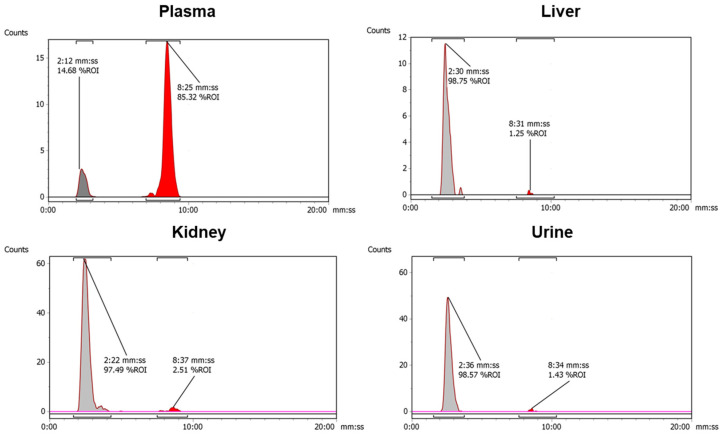
Representative radio-HPLC chromatograms of metabolites extracted from the plasma, liver, kidney, and urine at 30 min. Red peak at *t*_R_ = 8 min 27 s represents [^18^F]AlF-EMP-105.

Encouraged by the positive in vitro findings, we evaluated the in vivo chemical fate of [^18^F]AlF-EMP-105. Metabolite analysis was conducted at 5 and 30 min post-injection for the kidney, liver, blood plasma and urine (Table 2). The chemical forms of the accumulated radioactivity were identified by analytical radio-HPLC (Figure 4). 

[^18^F]AlF-EMP-105 remains largely intact in the blood plasma at 5 and 30 min p.i. (98.3 ± 1.1 and 93.3 ± 7.4%, respectively). However, in the kidney, liver and urine, almost none of the tracers remain, with a more polar radioactive species being detected at the solvent front (Figure 4). This polar species is likely to be free ^18^F^−^ or [^18^F]AlF^2+^, which co-eluted at the solvent front when the two analytes were injected onto the same RP-HPLC system.

### 3.4. In Vivo Kinetics and Biodistribution Studies

**Figure 5:**
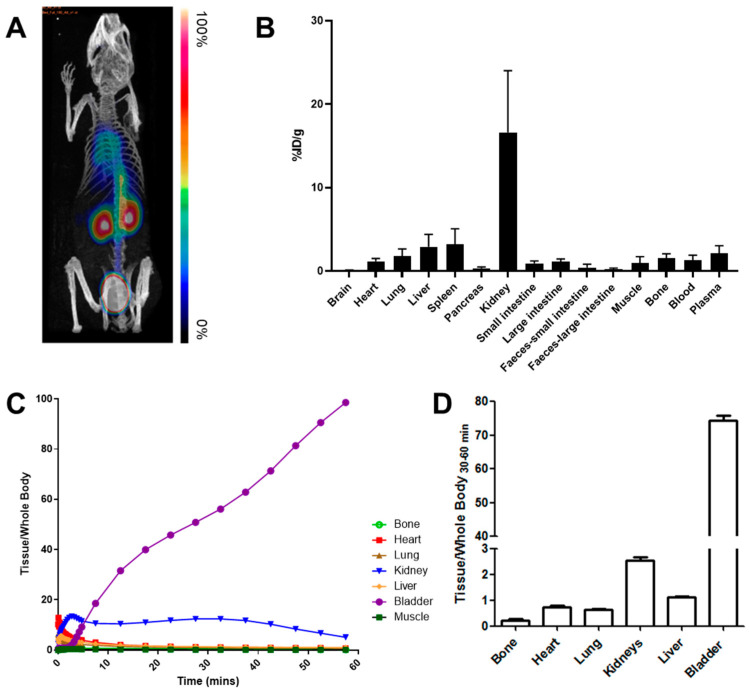
(**A**) Representative maximum intensity projection image of [^18^F]AlF-EMP-105 PET scan (fused for 60 min) after injection of 4.6 ± 1.2 MBq of the tracer via the tail vein. (**B**) Ex vivo biodistribution of [^18^F]AlF-EMP-105 in key organs 60 min p.i., excluding urine (428.1 ± 183.9 %ID/g) for clarity. (**C**) TACs of [^18^F]AlF-EMP-105 in vital organs. (**D**) Accumulation of radioactivity in key organs 30–60 min p.i. in vivo, normalized to whole-body activity. Data presented as mean ± SD, *n* = 3.

Dynamic PET imaging was performed on healthy mice for 60 min post-injection (Figure 5). The highest signal in the PET images was observed in the bladder, followed by the kidney. This was corroborated by ex vivo biodistribution studies, where the urine and kidney showed the largest concentration of radioactivity (428.1 ± 183.9 and 16.6 ± 7.4 %ID/g, respectively). Notably, only minor bone uptake was observed (1.6 ± 0.5 %ID/g), suggesting that the free ^18^F^−^ or [^18^F]AlF^2+^ produced does not re-enter the systemic circulation. Low liver uptake also signifies quantitatively limited uptake and transformation in this organ. 

The kinetic analysis of [^18^F]AlF-EMP-105 showed rapid tissue distribution, where activity in the heart, lung and liver peaked after 1 min (Figure 5C). Rapid clearance through the renal pathway was also observed, with the activity in the kidneys peaking at 2.5 min, and the bladder showing increasing uptake for the whole imaging period.

## 4. Discussion

With c-Met emerging as an important therapeutic and imaging target [1], we aimed to develop a facile method to obtain a ^18^F-labelled probe for c-Met imaging by PET. A peptide-based probe analogous to EMI-137 was designed due to the promising results obtained from human trials with both the fluorescent-labelled EMI-137 and ^68^Ga-labelled [^68^Ga]Ga-EMP-100 peptides [22,31]. Using the [^18^F]AlF method developed by McBride et al. [36,45], we successfully synthesised [^18^F]AlF-EMP-105 with a moderate RCY (46 ± 2%) and excellent RCP (>95%). Both the RCY and A_m_ obtained in this study were consistent with reported examples of [^18^F]AlF probes with NOTA chelators [36,46]. 

With [^18^F]AlF-EMP-105 showing excellent in vitro stability, the identification of c-Met-expressing cell lines (OE21, HT29 and H1975) and a negative control (HEPG2a) was carried out by flow cytometry. Subsequent radioactive in vitro uptake studies demonstrated that [^18^F]AlF-EMP-105 was specific for c-Met-expressing cells. This was shown through blocking studies with EMI-137, where OE21, HT29, and H1975 showed statistically significant decreases in the uptake of [^18^F]AlF-EMP-105. In contrast, this was not observed for HEPG2a. 

Metabolic analysis highlighted the in vivo chemical fate of [^18^F]AlF-EMP-105. It was found that [^18^F]AlF-EMP-105 showed high stability in the plasma, with 98% and 93% remaining intact after 5 and 30 min, respectively. Surprisingly, <5% of the tracer remained in the kidneys and liver after 5 min, where most of the accumulated radioactivity was found to be free ^18^F^−^ or [^18^F]AlF^2+^. This suggests that transchelation, demetallation and/or defluorination takes place in these tissues, causing the [^18^F]AlF-NOTA moiety to be transformed. Since the tracer does not show degradation when incubated with EDTA and human serum albumin, it can be inferred that these in vitro stability tests do not fully translate to in vivo conditions. Superoxide dismutase, caeruloplasmin and metallothionein, highly expressed in the hepatic and renal tissues, are candidate enzymes/proteins that can influence differences in the in vitro to in vivo kinetic stability.

In vivo and ex vivo analysis of the distribution of [^18^F]AlF-EMP-105 showed a high radioactive accumulation in the kidneys, bladder and urine. Thus, it is likely to be predominantly excreted by the renal pathway. The rapid distribution and low retention of [^18^F]AlF-EMP-105 in background organs is desirable in these studies, and the very high radioactivity in the bladder would perhaps mandate the future implementation of a bladder-voiding routine as part of imaging studies. Of note, in the present protocol, mice were anaesthetised throughout from injection through scanning, which affects the voiding of urine.

In comparison, the relatively low radioactive accumulation in the liver and gastrointestinal excretions indicates that only a small percentage of the tracer is cleared by the hepatobiliary system. The low bone uptake (1.6 ± 0.5 %ID/g) suggests that the tracer has high in vivo systemic stability, consistent with reported [^18^F]AlF tracers [36,42,45,47], where values of 0.4–1.0 %ID/g were observed for tracers using NOTA-derived chelators [36,48]. 

Although metabolite analysis revealed that ^18^F^−^ or [^18^F]AlF^2+^ were produced in the kidneys and liver, bone uptake remained low. This is despite the fact that ^18^F^−^ and [^18^F]AlF^2+^ are known to accumulate in bone [49,50,51]; McBride et al. showed that a nearly identical distribution results from the injection of [^18^F]AlF^2+^ or [^18^F]F^−^, with both species showing uptake in the spine ([^18^F]F^−^: 19.03 %ID/g, [^18^F]AlF^2+^ 19.88 %ID/g) [36]. One possible explanation is that the ^18^F^−^ or [^18^F]AlF^2+^ produced remains trapped in the respective tissues, and was excreted without re-introduction into the systemic circulation. Given the low radioactive uptake of [^18^F]AlF-EMP-105 in the liver, it could be said that the extent and rate of transformation of the tracer in this organ is quantitatively limited. In comparison, the high radioactive accumulation in the kidneys and bladder indicates that for [^18^F]AlF-EMP-105, radioactivity is mainly excreted in the form of ^18^F^−^ and/or [^18^F]AlF^2+^ via the urinary pathway.

Whilst we have not conducted a head-to-head comparison with [^68^Ga]Ga-EMP-100, there are some general advantages of [^18^F]AlF-EMP-105 compared to its gallium-68 analogue (same binding peptide). With a longer physical half-life and wider network of cyclotrons manufacturing high gigabecquerel activity of fluorine-18, it is envisaged that [^18^F]AlF-EMP-105 will be a candidate for decentralized manufacture, making it more accessible for routine clinical use. Furthermore, the lower positron energy of fluorine-18 (0.65 MeV) is at least theoretically advantageous in relation to a spatial resolution compared to much energetic gallium-68 (1.90 MeV).

One limitation of this work is that the in vivo specificity and performance of [^18^F]AlF-EMP-105 for c-Met imaging was not evaluated. Thus, future work will focus on determining the efficacy of the tracer for detecting c-Met in vivo using suitable tumour models. Another possible improvement would be to use the pentadentate 1,4,7-triazacyclononane-1,4-diacetate (NODA) chelator instead of the hexadentate 1,4,7-triazacyclononane-1,4,7-triacetate (NOTA) chelator [45,48], which has been proven to increase radiochemical yield to >95%. This is because the coordination sphere of the [^18^F]AlF^2+^ complex would be completed by 5 additional donors, whereas a 6th donor arm on NOTA would compete with the [^18^F]fluoride, decreasing reaction yield. 

Automation of the radiosynthesis of [^18^F]AlF-EMP-105 could also be attempted, which can produce a larger radioactive dose of the tracer and increase its molar activity. The stability of [^18^F]AlF-EMP-105 to radiolysis could also be evaluated at higher radioactive concentrations.

## 5. Conclusions

We report the development of a facile and convenient method to access an ^18^F-labelled tracer for imaging c-Met. Preliminary biological evaluations showed that [^18^F]AlF-EMP-105 binds specifically to a diverse range of c-Met-expressing cells in vitro, and has sufficient blood serum stability ex vivo and in vivo for c-Met imaging. These findings show that [^18^F]AlF-EMP-105 is a promising alternative to [^68^Ga]Ga-EMP-100. Future work would include validating the diagnostic performance of [^18^F]AlF-EMP-105 using suitable tumour models in vivo.

## Figures and Tables

**Table 1:** Optimisation of [^18^F]AlF-labelling of EMP-105 based on precursor amount, where the synthesis required 35–40 min. Data are presented as mean ± s.d., *n* = 3.

Precursor Amount (nmol)	Radiochemical Conversion (%) ^a^	Isolated Activity (MBq)	Radiochemical Yield (%) ^b^	Molar Activity (GBq/µmol)
20	30 ± 3	48 ± 8	20 ± 3	2.4 ± 0.4
50	56 ± 4	153 ± 20	46 ± 2	3.3 ± 0.5
100	58 ± 2	168 ± 8	50 ± 5	1.7 ± 0.1

^a^ Determined by radio-HPLC. ^b^ Decay corrected to the start of synthesis.

**Table 2:** Metabolite analysis in key tissues, showing percentage of [^18^F]AlF-EMP-105 remaining after 5 and 30 min post injection. No urine sample was obtained at 5 min p.i. Data reported as mean ± s.d., *n* = 3.

	Percentage of [^18^F]AlF-EMP-105 Remaining (%)		Extraction Efficiency (%)	
Tissue	5 min p.i.	30 min p.i.	5 min p.i.	30 min p.i.
Plasma	98.3 ± 1.1	93.3 ± 7.4	50.1 ± 13.4	42.9 ± 25.4
Kidney	9.8 ± 12.9	4.3 ± 4.3	56.1 ± 25.9	47.7 ± 31.2
Liver	6.2 ± 10.8	0.4 ± 0.7	25.6 ± 11.7	31.9 ± 12.7
Urine	-	7.9 ± 9.2	-	-

## Data Availability

The data generated in this study are included in this published article or the associated supplementary information file.

## Supplementary Materials

The following supporting information can be downloaded at: https://www.mdpi.com/article/10.3390/pharmaceutics15071915/s1, Figure S1: Reference UV-HPLC chromatogram of NOTA-EMP-105; Figure S2: Radio-HPLC chromatogram of purified [^18^F]AlF-NOTA-EMP-105; Figure S3: Stacked radio-HPLC chromatograms of [^18^F]AlF-NOTA-EMP-105 after incubation in PBS, human serum, RPMI media and EDTA at 37 °C; Figure S4: Stacked radio-HPLC chromatograms of >2 GBq of [^18^F]AlF-NOTA-EMP-105; Figure S5: Calibration curve for molar activity calculation, obtained using EMP-105. Data expressed as mean of 3 replicates; Table S1: Percentage of [^18^F]AlF-NOTA-EMP-105 remaining after incubation in at 37 in PBS, human serum, RPMI media and EDTA °C.

## Abbreviations

A_m_	Molar activity
BCA	Bicinchoninic acid assay
DMSO	Dimethyl sulfoxide
RCC	Radiochemical conversion
RCP	Radiochemical purity
RCY	Radiochemical yield
MeCN	Acetonitrile
NODA	1,4,7-triazacyclononane-1,4-diacetate
NOTA	1,4,7-triazacyclononane-1,4,7-triacetate
PET	Positron emission tomography
RIPA	Radioimmunoprecipitation assay buffer
*t*_R_	Retention time
SD	Standard deviation
SEM	Standard error of the mean
TFA	Trifluoroacetic acid
