# Supplementary material for: Evaluation of [18F]AlF-EMP-105 for Molecular Imaging of C-Met
Source: Pharmaceutics. 2023 Jul 10;15(7):1915. doi: 10.3390/pharmaceutics15071915 (PMC10383791; doi:10.3390/pharmaceutics15071915)
Supplement: Supplementary file 1 [file pharmaceutics-15-01915-s001.zip › pharmaceutics-2428469-supplementary.pdf]

Supplementary Information

Evaluation of [^{18}F]AlF-NOTA-EMP-105 for Molecular Imaging of C-Met

Jin Hui Teh [1,†], Ala Amgheib [2,†], Ruisi Fu [2], Chris Barnes [2], Joel Abrahams [2], Ali Ashek [2], Ning Wang [2], Zixuan Yang [2], Muneera Mansoorudeen [2], Nicholas J. Long [1] and Eric O. Aboagye [2,*]

[1] Department of Chemistry, Molecular Sciences Research Hub, Imperial College London, London W12 0BZ, UK; jin.teh15@imperial.ac.uk (J.H.T.); n.long@imperial.ac.uk (N.J.L.)
[2] Department of Surgery and Cancer, Imperial Centre for Translational and Experimental Medicine, Imperial College London, London W12 0NN, UK; a.amgheib@imperial.ac.uk (A.A.); ruisi.fu14@imperial.ac.uk (R.F.); chris.barnes@imperial.ac.uk (C.B.); joel.abrahams@imperial.ac.uk (J.A.); m.ashek@imperial.ac.uk (A.A.); n.wang16@imperial.ac.uk (N.W.); zixuan.yang18@imperial.ac.uk (Z.Y.); m.mansoorudeen22@imperial.ac.uk (M.M.)
* Correspondence: eric.aboagye@imperial.ac.uk
† These authors contributed equally to this work.

Radiochemistry

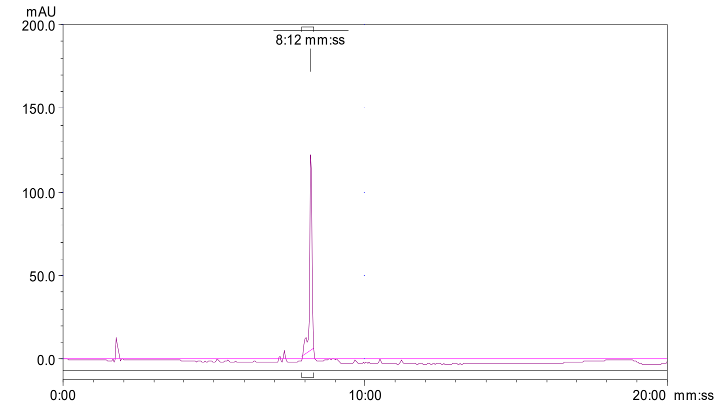

Figure S1: Reference UV-HPLC chromatogram of NOTA-EMP-105

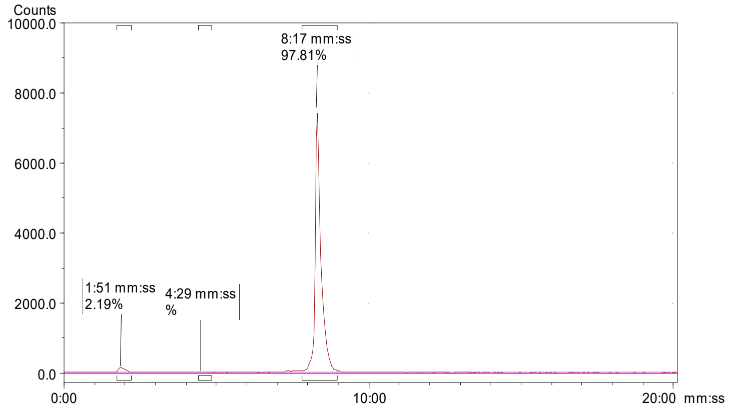

Figure S2: Radio-HPLC chromatogram of purified [^{18}F]AlF-NOTA-EMP-105

In vitro stability studies

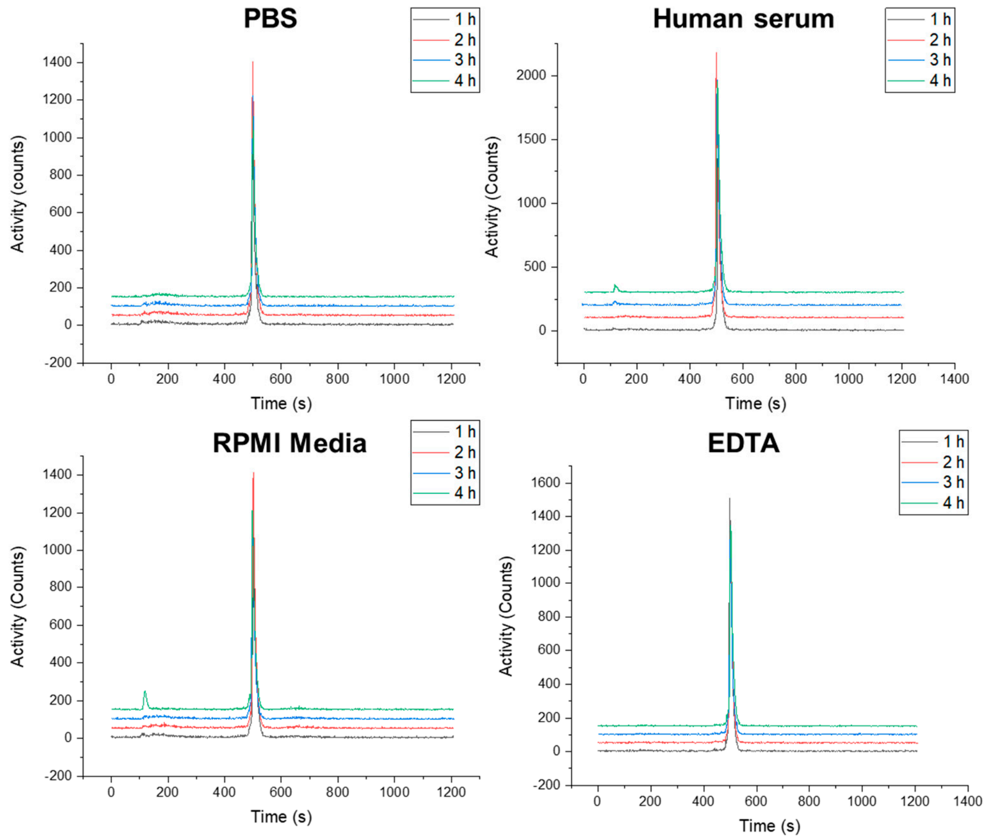

Figure S3: Stacked radio-HPLC chromatograms of [^{18}F]AlF-NOTA-EMP-105 after incubation in PBS, human serum, RPMI media and EDTA at 37 ^{o}C

Table S1: Percentage of [^{18}F]AlF-NOTA-EMP-105 remaining after incubation in at 37 in PBS, human serum, RPMI media and EDTA ^{o}C.

Time (h)	PBS (%)	Human Serum (%)	RPMI (%)	EDTA (%)
1	98	99	97	99
2	97	99	97	99
3	95	98	97	98
4	95	97	92	98

Radiolysis tests

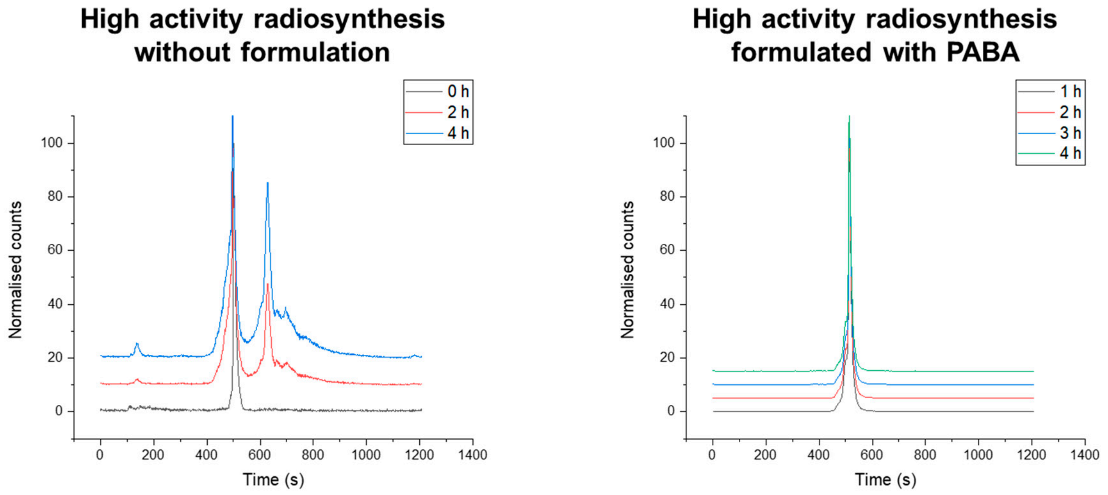

Figure S4: Stacked radio-HPLC chromatograms of >2 GBq of [^{18}F]AlF-NOTA-EMP-105: Left: eluted from cartridge or; Right: formulated in PBS solution containing 5 mg/mL (10 mL) of PABA (4-aminobenzoic acid) and 7.5% EtOH.

Calibration curve for molar activity determination

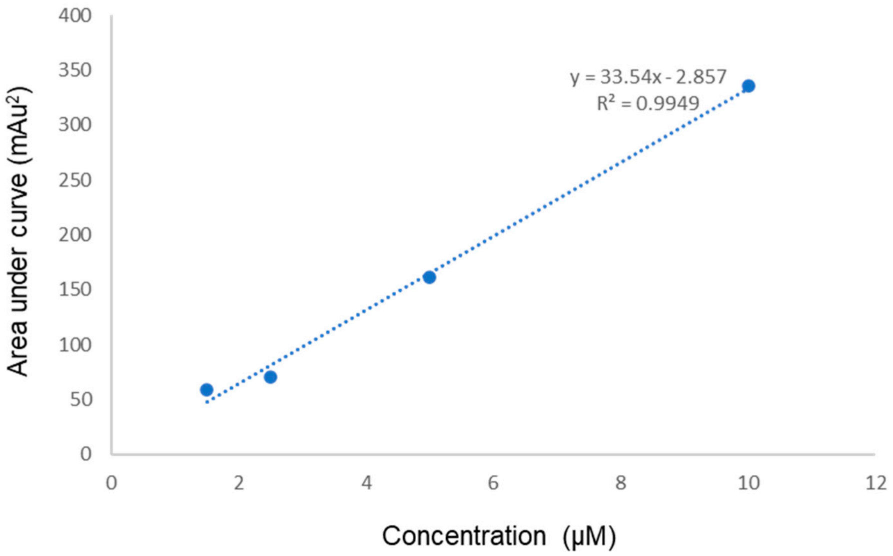

Figure S5: Calibration curve for molar activity calculation, obtained using EMP-105. Data expressed as mean of 3 replicates.
